# Prognostic Value of Preoperative Echocardiographic Findings in Patients Undergoing Transvenous Lead Extraction

**DOI:** 10.3390/ijerph18041862

**Published:** 2021-02-14

**Authors:** Dorota Nowosielecka, Wojciech Jacheć, Anna Polewczyk, Łukasz Tułecki, Andrzej Kleinrok, Andrzej Kutarski

**Affiliations:** 1 Department of Cardiology, The Pope John Paul II Province Hospital, 22-400 Zamość, Poland; dornowos@wp.pl (D.N.); a.kleinrok@wp.pl (A.K.); 2 2nd Department of Cardiology, Silesian Medical University, 40-055 Zabrze, Poland; wjachec@interia.pl; 3 Department of Physiology, Pathophysiology and Clinical Immunology, Collegium Medicum, Jan Kochanowski University, 25-369 Kielce, Poland; 4 Department of Cardiac Surgery, Świętokrzyskie Cardiology Center, 25-001 Kielce, Poland; 5 Department of Cardiac Surgery, The Pope John Paul II Province Hospital, 22-400 Zamość, Poland; luke27@poczta.onet.pl; 6 Department of Physiotherapy, Medical College of University of Technology and Management, 35-225 Rzeszów, Poland; 7 Department of Cardiology, Medical University, 20-059 Lublin, Poland; Andrzej.Kutarski@ptkardio.Lublin.pl

**Keywords:** transesophageal echocardiography, vegetations, tricuspid valve dysfunction, transvenous lead extraction, long-term survival

## Abstract

(1) Background: In patients referred for transvenous lead extraction (TLE) transesophageal echocardiography (TEE) often reveals abnormalities related to chronically indwelling endocardial leads. The purpose of this study was to determine whether the results of pre-operative TEE might influence the long-term prognosis. (2) Methods: We analyzed data from 936 TEE examinations performed at a high volume center in patients referred for TLE from 2015 to 2019. The follow-up was 566.2 ± 224.5 days. (3) Results: Multivariate analysis of TEE parameters showed that vegetations (HR = 2.631 [1.738–3.983]; *p* < 0.001) and tricuspid valve (TV) dysfunction unrelated to the endocardial lead (HR = 1.481 [1.261–1.740]; *p* < 0.001) were associated with increased risk for long-term mortality. Presence of fibrous tissue binding sites between the lead and the superior vena cava (SVC) and/or right atrium (RA) wall (HR = 0.285; *p* = 0.035), presence of penetration or perforation of the lead through the cardiac wall up to the epicardium (HR = 0.496; *p* = 0.035) and presence of excessive lead loops (HR = 0.528; *p* = 0.026) showed a better prognosis. After adjustment the statistical model with recognized poor prognosis factors only vegetations were confirmed as a risk factor (HR = 2.613; *p* = 0.039). A better prognosis was observed in patients with fibrous tissue binding sites between the lead and the superior vena cava (SVC) and/or right atrium (RA) wall (HR = 0.270; *p* = 0.040). (4) Conclusions: Non-modifiable factors may have a negative influence on long-term survival after TLE. Various forms of connective tissue overgrowth and abnormal course of the leads modifiable by TLE can be a factor of better prognosis after TLE.

## 1. Introduction

Recently, due to the rising incidence of infectious and non-infectious complications related to cardiac implantable electronic devices (CIED), the number of transvenous lead extraction (TLE) procedures has also been increasing [1]. TLE is considered as a first-line strategy for the management of CIED-associated complications [2,3]. The rate of major complications associated with TLE has been estimated to range from 0.9 to 4.0%, and most often there is damage to the heart or venous vessels; the lead extraction procedure carries a 0 to 0.4% risk of death [2,3]. Due to the continuous improvement in the extraction strategy, most patients with major complications are discharged from hospital in a good general state [4]. Therefore, theoretically the fate of patients after TLE should not differ from those who did not undergo TLE. There is a large volume of published studies describing TLE outcomes, however the results are still unsatisfactory, because mortality is 5–25% at one year, 8–38% at three years, 8–44% at 5 years and 10–60% at 10 years, with the lowest values encountered in patients with non-infectious indications and highest in those with lead-related infective endocarditis (LRIE) [5,6,7,8,9,10,11,12,13,14,15,16,17,18,19,20,21,22,23]. Previous studies have not analyzed the effect of echocardiographic phenomena on long-term survival of patients undergoing TLE, and few reports have only assessed their relationship with the risk of procedure. The main echocardiographic parameter considered in order to estimate the risk of surgery was the value of the left ventricular ejection fraction (LVEF) [6,7,12,16] as well as the presence/size of vegetation [7,9,10,11,13,14,15,18,19]. Only a few studies based on small sample sizes considered a possible impact of asymptomatic masses on endocardial leads (AMEL) on the length of survival following TLE [24,25,26,27,28,29]. This paper provides an in-depth analysis of preoperative TEE findings and their usefulness for predicting long-term outcomes of TLE.

## 2. Methods

### 2.1. Study Population

A prospective analysis was carried out on data from preoperative TEE performed at a high-volume center during 936 TLE procedures from June, 2015 to October 2019. All patients gave their written informed consent to TLE and analysis of anonymized medical records, approved by the Bioethics Committee of the Regional Chamber of Physicians and Dentists in Lublin no. 288/2018/KB/VII.

### 2.2. Factors Potentially Affecting Long-Term Survival after TLE

In order to identify the factors that may influence long-term survival the following variables have been analyzed:

Patient-dependent factors: age (during TLE and at first CIED implantation), gender, NYHA class, LVEF, atrial fibrillation, chronic renal failure, diabetes, arterial hypertension, a history of coronary artery bypass graft (CABG), previous sternotomy, CHA2DS2-VAsc score, Charlson comorbidity index, chronic anticoagulation, and antiplatelet therapy.

CIED-related factors: the number of leads in the device before TLE, the number of leads the patient had before TLE, abandoned leads, excessive lead loops before TLE, high voltage (HV) leads, leads in the coronary sinus (CS), dwell time of the oldest lead in the patient, mean implant duration before TLE, cumulative dwell time of the extracted leads, and the number of CIED procedures before TEE.

Indication-related data: diagnosis of LRIE certain or probable with or without pocket infection or only local pocket infection.

TLE efficacy and complications: the rate of complete radiographic success, partial radiographic success, lack of radiographic success, clinical success, complete procedural success and presence of any major complication, hemopericardium, severe tricuspid valve damage during TLE, rescue cardiac surgery.

Most important preoperative TEE findings: tricuspid valve dysfunction, lead-dependent tricuspid valve dysfunction (LDTD), shadowing from the leads before TLE, fibrous tissue binding the lead to the heart structures, AMEL (fibrous tissue encasing the lead, lead thickening, clots and vegetation-like masses), vegetations, excessive lead loops and perforation or penetration of the lead through the cardiac wall up to the epicardium.

### 2.3. Lead Extraction Procedure

TLE was defined according to EHRA consensus document as intervention with removal of at least one lead that has been implanted for more than one year or a lead regardless of duration of implant requiring the assistance of specialized equipment that is not included as part of the of the typical implant package and/or removal of a lead from a route other than the implant vein [30].

Complete procedural success was defined as removal of all targeted leads and material, with the absence of any permanently disabling complication or procedure-related death. Clinical procedural success was defined as retention of a small portion of a lead (<4 cm) that does not negatively impact the outcome goals of the procedure and with absence of any permanently disabling complication or procedure-related death [30].

Extraction procedures were performed in a hybrid operating room or in an operating room, using mechanical systems such as polypropylene Byrd dilators (Cook^®^ Medical, Leechburg, PA, USA), making use of the oblique cutting edge of the tip to dissect leads from fibrous sheaths that immobilized the lead in the intravascular and/or intracardiac segment [11,28]. Procedures were performed in patients under general anesthesia and after preparation of the surgical field as for subjects coming in for cardiac surgery. Continuous invasive blood pressure monitoring from radial artery was used. The composition of the surgical team and the course of the extraction procedure have been described in detail elsewhere [31,32,33].

### 2.4. Preoperative TEE

TEE was performed using the Philips iE33 (Phillips Healthcare, Andover, MA, USA) or the GE Vivid S70 (General Electric Company, Boston, MA, USA) ultrasound machine equipped with X7-2t Live 3D or 6VT-D probes. Images and recordings were obtained before the procedure, after general anesthesia and tracheal intubation, during preparation of the surgical field, and dissection and stabilization of the leads in the region of the device pocket. Leads were evaluated in the mid-esophageal, inferior esophageal and modified transgastric views to visualize the right heart chambers and the tricuspid valve. In order to obtain complete visualization of the structures (and assessment of lead/heart interface) non-standard imaging planes were sometimes required. After the procedure the results were entered into a computer database. The TEE examination was described in detail in previous publications- we followed the methods of Nowosielecka et al. [31,32,33].

### 2.5. Echocardiographic Findings Associated with Endocardial Leads: Definition and Classification According to the Anatomy and Characteristic Features

**Figure 1:**
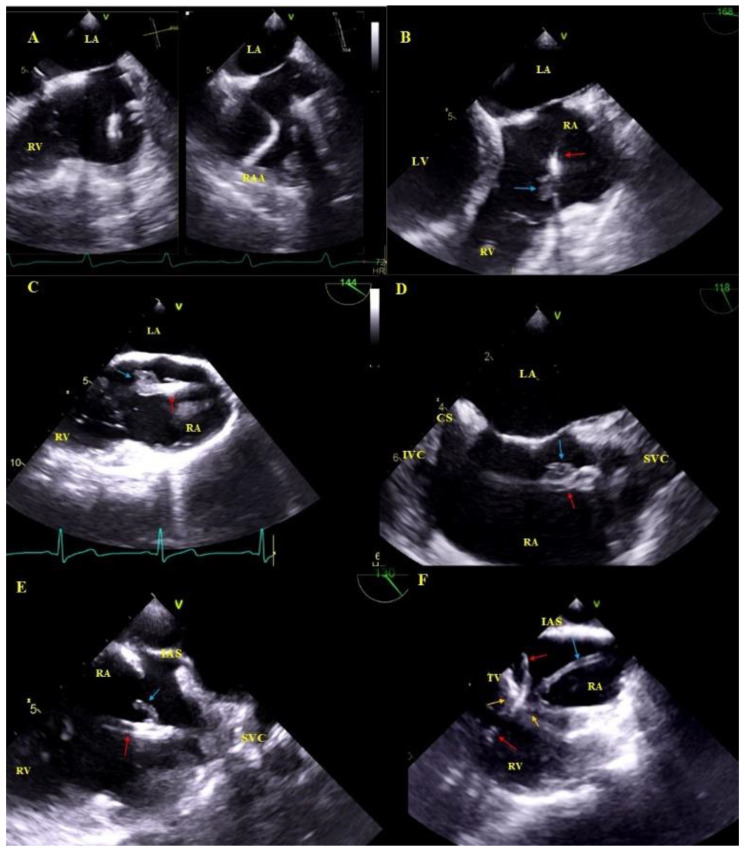
Asymptomatic masses on endocardial leads in 2D TEE. (**A**)—Thickened hyperechoic distal segment of atrial lead surrounded by a connective tissue sheath. (**B**)—Thickened hyperechoic segment of atrial lead (red arrow) with a mobile mass representing a clot (blue arrow). (**C**,**D**)—Ventricular lead (red arrow) in the RA with mobile vegetation-like masses (blue arrow) (2D, ME modified and bicaval). (**E**)—Ventricular lead (red arrow) in the RA with a mobile connective tissue mass (accretion) (blue arrow). (**F**)—The thickened ventricular lead adhered to the TV leaflets, in addition, in RA, the echo associated with the TV (blue arrow) is the sheath of silicone insulation remaining after the first TLE of removing the previous ventricular lead. The place of growth is marked with yellow arrows.

Asymptomatic masses on endocardial leads (AMEL) [31]: Additional masses on the leads classified as clots (varying degrees of organization), components of connective tissue (so-called accretions), masses resembling vegetations (vegetation-like masses), probably the remnants after infections: Old fibrous vegetations or clots (Figure 1).

**Figure 2:**
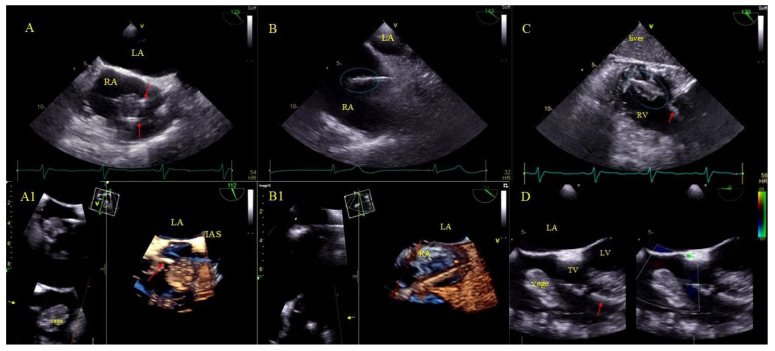
LRIE—TEE images of bacterial vegetations attached to CIED leads. (**A**,**A1**)—Bacterial vegetation attached to the lead (red arrows) in the RA in 2D and 3D TEE (bicaval). (**B**,**B1**)—Fine vegetations on the lead causing lead thickening with irregular contour (blue circle) in 2D TEE (**B**)—well visible in 3D TEE (**B1**)—(bicaval). (**C**)—Echoes of the lead (red arrow) with vegetations (blue circle) in the RV (2D, TG, TEE). (**D**)—Large bacterial vegetation attached the ventricular lead (red arrow) dislodging to the TV orifice without significant impact on valve function. The size of the vegetation disqualifies the patient from TLE (2D, 4-CH TEE).

Bacterial vegetations [31], i.e., multishaped, mobile masses of inhomogeneous echogenicity. Vegetations were diagnosed only if they were accompanied by signs of a general infection (positive inflammatory markers, positive blood cultures) or a regional infection (pocket infection) (Figure 2).

Hyperechoic segmental thickening of the leads defined as connective tissue overgrowth (undergoing fibrosis, mineralization, crystallization and even ossification) [31].

**Figure 3:**
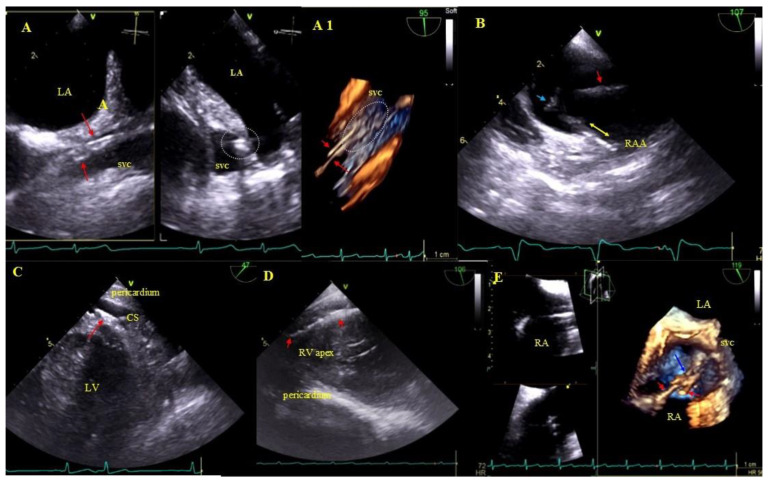
Lead adhesion in various parts of the cardiovascular system. (**A**,**A1**)—Two leads (red arrows) bound together and adhering to the SVC wall (2D and 3D, bicaval). (**B**)—The end of the atrial lead (red arrow) implanted in the RAA adhering to the RA wall (yellow arrow) and a mass on the lead (accretion) (blue arrow) (2D, bicaval, modified). (**C**)—In the coronary sinus the end of the lead adhering to the vascular wall (red arrow) (2D, TG modified). (**D**)—Long distal end of the ventricular lead (red arrows) adhering to the RV endocardium (2D, TG). (**E**)—In the RA an additional leads (red arrows) fibrous mass (accretion) (blue arrow) at the binding site (3D, bicaval).

Buildup: Fibrous connective tissue sheath around the lead causing adherence to the endocardium and vessel walls producing images similar to segmental lead thickening but moving along with the cardiac wall. The term encompasses also segmental lead-to-lead adhesion (two or three leads) moving along together with the cardiac walls. Immobile masses binding the lead to the vein or heart wall most frequently represent a sign of pre-existing asymptomatic inflammatory response triggered by the endocardial lead (foreign body reaction). Over time, fibrosis ensues with the presence of calcifications (mineralization, crystallization and ossification). This type of reaction may occur in patients with and without device infections [31] (Figure 3).

**Figure 4:**
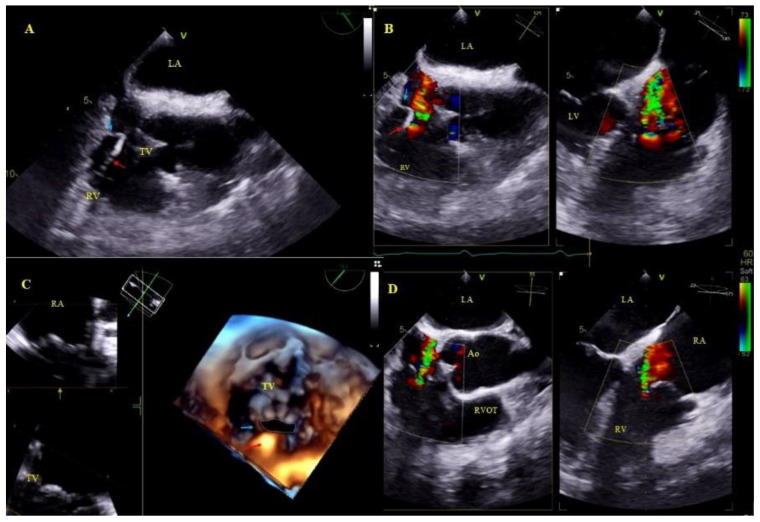
Lead-dependent tricuspid dysfunction (LDTD) due to lead impingement (**A**)—The lead (red arrow) impinging on the posterior TV leaflet (blue arrow) (2D, ME). (**B**)—Color Doppler shows severe tricuspid regurgitation before TLE (red arrow-lead, blue arrow- posterior TV leaflet). (**C**)—3D TEE viewed from the RV- impinging on the posterior TV leaflet (red arrow-lead, blue arrow- posterior TV leaflet). (**D**)—Moderate tricuspid regurgitation after the extraction procedure (2D, color Doppler).

Other, separately classified lead-associated phenomena: Lead-dependent tricuspid dysfunction: valve regurgitation (very rarely TV stenosis) unquestionably caused by the lead (lead impingement, lead entanglement with tendinous chords, lead adhesion to the leaflet, leaflet perforation) [31] (Figure 4).

**Figure 5:**
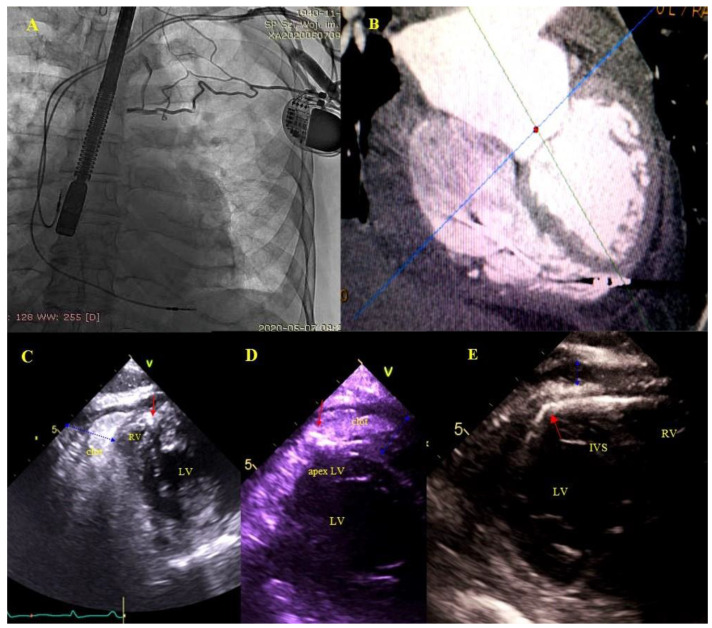
Right ventricular wall perforation by the ventricular lead. An 80-year-old patient with a DDD pacemaker and recurrent pericardial effusion for 3 months. Based on the location of the ventricular lead tip on chest X rays (**A**)—and TEE, perforation of the RV wall was suspected. ECG-gated CT confirmed the diagnosis (**B**)—TEE (2D, TG) during the procedure visualized the end of the perforating lead (red arrows) (**C**–**E**)—and a clot in the pericardium (blue arrow) (**C**,**D**).

Cardiac wall perforation by the lead: visualization of the lead tip outside the heart contour, frequently with fluid in the pericardial sac; placement of the lead tip close to the border of the pericardium is referred to as penetration (Figure 5).

**Figure 6:**
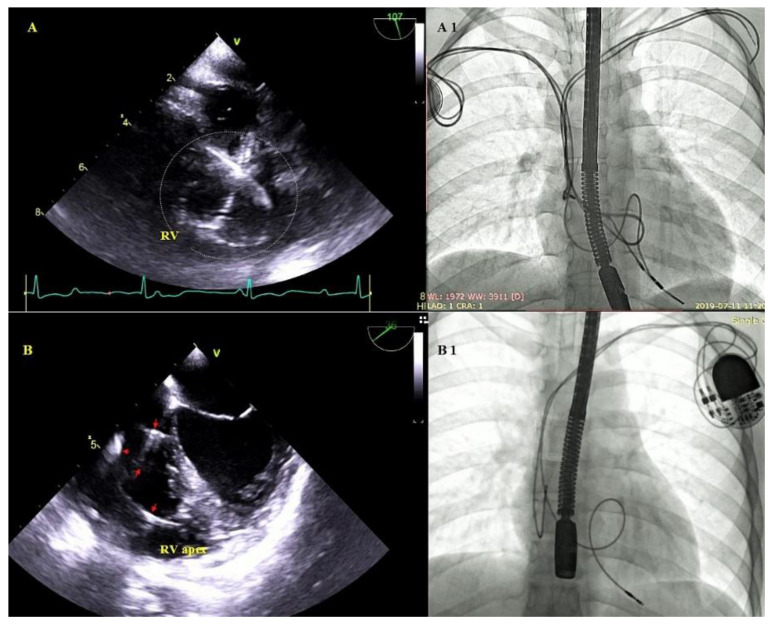
Excessive lead loops in TEE and fluoroscopy during TLE. (**A**,**A1**)—Excessive loops of ventricular and atrial leads (white circle) visualized in the RV cavity with multifocal lead-to-lead binding. (**B**,**B1**)—Excessive loop of the ventricular lead (red arrows) in the RV cavity.

Excessive lead loops as a result of too weak fixation during implantation or lead fracture with insulation breach in the subclavian region. Excessive lead loops may be encountered in the right atrium or the right ventricle, and in the tricuspid valve orifice (Figure 6).

## 3. Statistical Analysis

The Shapiro–Wilk test was used to test the distribution of continuous variables. A non-parametric distribution of all continuous variables was found. For a clearer presentation of the results, all continuous variables are presented as the mean ± standard deviation and some of them (patient age during TLE and during first CIED implantation, left ventricle ejection fraction, dwell time of the oldest lead in the patient) additionally as median with the first and the third quartile. The categorical variables are presented as number and percentage. The study population was divided into two groups depending on TLE outcomes (survival versus death) at two-year follow-up. The significance of differences between the groups was determined using the nonparametric “U” Mann–Whitney test and Chi square tests. The relationship between the echocardiographic parameters and mortality after TLE was analyzed using Cox regression analysis. All variables reached *p* < 0.1 in univariate analysis were included into a multivariate model. Two multivariate regression models were defined. Model 1 was built to assess the prognostic value of the echocardiographic variables only. Model 2 included echocardiographic variables from model 1 and adjusted by clinical parameters of known prognostic values (patient age at first implantation, patient age during TLE, gender, LVEF, NYHA functional class, presence of diabetes mellitus, renal failure, arterial hypertension, infectious indications for TLE, ICD and CRTD prior to TLE). Moreover, the impact of the binding sites between leads and VCS wall and/or right atrial wall and survival after TLE was presented as the Kaplan-Meier survival curves. The log rank test was used to compare the survival distributions of the groups. A two-tailed *p* value < 0.05 was considered statistically significant.

Statistical analysis was performed with STATISTICA 13.0 (TIBCO Software Inc. Krakow, Poland). All patients gave informed consent for TLE and anonymous analysis of their medical records, approved by the local Bioethics Committee.

## 4. Results

Transesophageal echocardiography before TLE was performed in 936 patients (355 women; 37.93%), with a mean age of 67.08 ± 14.50 years. The indications for TLE were mainly noninfectious (727 patients; 77.67%). Pocket infection was recognized in 58 (6.20%) patients, whereas lead-related infective endocarditis in 151 (16.13%) individuals. The follow-up after TLE was 566.2 ± 224.5 days (range: 2–730). There were 112 deaths during follow-up. Patients with infectious indications for TLE, especially with LRIE, had a worse survival compared to patients with non-infectious indications: 559.4 ± 266.8 vs. 670.6 ± 167.8 days; *p* < 0.001 (the time of survival during follow-up were calculated for patients with completed two-years follow-up, *n* = 612).

### 4.1. Prognostic Factors

#### 4.1.1. Prognostic Factors Not Related to TEE Findings and TLE Procedure

Most of these deaths were attributed to patient-dependent risk factors: older age at first CIED implantation (*p* < 0.001), older age during TLE (*p* < 0.001), male gender (*p* = 0.003), higher NYHA class (*p* < 0.001), low LVEF (*p* < 0.001), atrial fibrillation (*p* < 0.001), chronic renal failure (increased, creatinine concentration >1.3 mg/dl) (*p* < 0.001), higher CHA2DS2-VAsc score (increased, *p* < 0.001), higher Charlson comorbidity index (*p* < 0.001) and chronic anticoagulation therapy (*p* < 0.001) (Table 1).

#### 4.1.2. Prognostic Factors Related to Implanted Devices

Of CIED-related factors, HV therapy (ICD lead presence) (*p* = 0.016), leads in the CS (LV pacing) (*p* < 0.001) and a higher number of leads the patient had before TLE (*p* = 0.029) were associated with lower survival rates. Similarly, infectious indications for TLE (LRIE) were associated with worse long-term survival (*p* < 0.001) (Table 1).

#### 4.1.3. Prognostic Factors Related to TLE Procedure

The factors related to procedure efficacy and major complications did not affect significantly long-term outcomes after TLE (Table 1).

#### 4.1.4. Prognostic Factors Related to TEE Findings

Of preoperative TEE variables, tricuspid valve dysfunction (degree of regurgitation) (*p* < 0.001) and vegetations (*p* < 0.001) were significantly more common among those who died after TLE. In contrast, the signs of connective tissue overgrowth occurred significantly more often among those who survived: fibrous tissue binding the lead to the superior vena cava and heart structures (*p* = 0.024), fibrous tissue binding the lead to the heart structures (all) (*p* = 0.008), fibrous tissue binding the lead to the RA wall (*p* < 0.001), lead-to-lead adhesion (*p* < 0.009), asymptomatic masses on endocardial leads (all) (*p* = 0.022), and fibrous tissue encasing the lead (*p* = 0.021). Similarly, the presence of (any) lead loops in the heart before TLE (*p* = 0.037) and perforation or penetration of the lead through the cardiac wall up to the epicardium (*p* = 0.038) were associated with better chances of long-term survival (Table 2).

### 4.2. Cox’s Regression Analysis Results (Model-1; Echocardiographic Data)

Univariate Cox regression analysis showed a negative relationship between the chances of two-year survival and disease-related parameters only: lead-unrelated TV dysfunction (HR = 1.528; *p* < 0.001) and vegetations (HR = 3.078; *p* < 0.001). However, on the other hand, there was a positive relationship between the chances of two-year survival and the following variables (related to implant duration): fibrous tissue encasing the lead (HR = 0.442; *p* = 0.014), fibrous tissue binding the lead to the SVC and RA wall (HR = 0.208; *p* = 0.007), and lead-to-lead adhesion (HR = 0.484; *p* = 0.022). Additionally, perforation or penetration of the lead through the cardiac wall up to the epicardium (HR = 0.474; *p* = 0.024) and excessive lead loops (HR = 0.543; *p* = 0.033) were suggestive of better prognosis (Table 3).

Multivariable Cox regression analysis of TEE variables confirmed the negative relationship between the chances of two-year survival and lead-unrelated TV dysfunction (HR = 1.481; *p* < 0.001) and vegetations (HR = 2.631; *p* < 0.001), and the positive relationship between fibrous tissue binding the lead to the SVC and/or RA wall (HR = 0.285; *p* = 0.036), perforation or penetration of the lead through the cardiac wall up to the epicardium (HR = 0.469; *p* = 0.035) and excessive lead loops (HR = 0.528; *p* = 0.026) (Table 3).

### 4.3. Cox’s Regression Analysis Results (Model-2; Echocardiographic Data Adjusted with Recognized Clinical Risk Factors)

**Figure 7:**
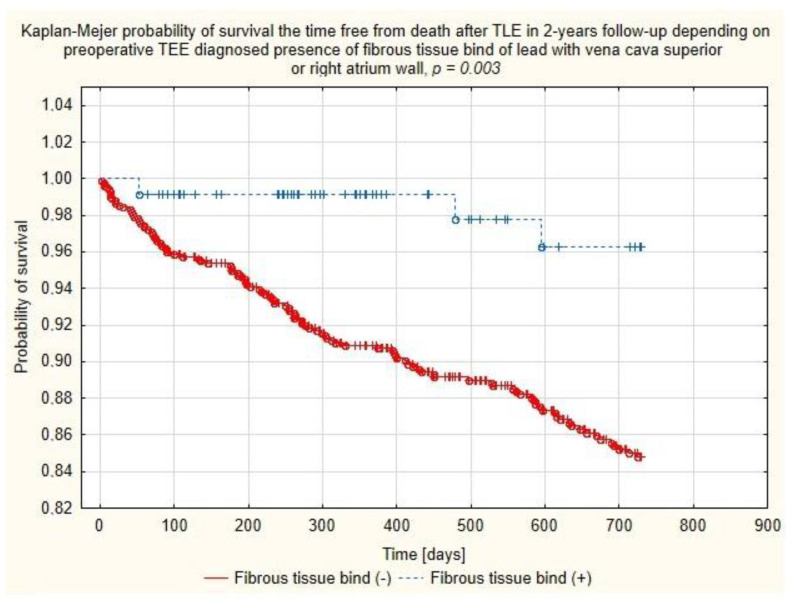
Kaplan–Meier probability of survival after TLE depending on the presence of binding sites between the lead and walls of the superior vena cava or right atrium.

Multivariate Cox regression analysis confirmed the negative relationship between the chances of two-year survival and patient’s health status parameters such as patient age during TLE (HR = 1.037, *p* = 0.057), decreased LVEF (per ↓10%p) (HR = 1.168, *p* = 0.051), the presence of chronic renal failure (HR = 1.811; *p* = 0.004), the lead in the CS before TLE (HR = 1.610; *p* = 0.031), long-term anticoagulation (HR = 1.550, *p* = 0.032) and indication-related parameters: vegetations (HR = 2.613; *p* < 0.001) and lead-related infective endocarditis (LRIE) without vegetations (HR = 2.371; *p* < 0.017). But, on the other hand, the analysis showed several variables predicting significantly better TLE outcomes, i.e., presence of fibrous tissue binding the lead to the SVC and/or RA wall (HR = 0.270; *p* = 0.040) and unexpectedly, arterial hypertension (HR = 0.569; *p* = 0.006). (Table 4 and Figure 7).

Results of multivariate model-2 Cox regression analysis are also presented in the Figure S1.

## 5. Discussion

Predicting long-term survival after various procedures, especially those related to the cardiovascular system, is an extremely important element of planning a therapeutic strategy. Transvenous lead extraction has been performed for a relatively short time, and for this reason only few studies have looked at the long-term prognosis of patients after TLE. The available evidence shows that CIED-related infection is the most common prognostic factor for unfavorable outcomes of TLE [5,6,7,8,9,10,11,13,14,15,17,18,19,20,21,22]. Other factors are mainly those dependent on the patient’s general condition i.e., age [6,13,21,22], renal failure [5,6,7,8,10,12,13,16,19,20,21,22], diabetes [5,16,21], heart failure [7,10,16,22], anemia [8,19], comorbidities [7]. Several studies have demonstrated the significance of procedure-related factors (system upgrade, ICD or CRT device, procedural failure, retained lead fragments, major complications, abandoned leads) [5,6,11,12,13,14,16,21] (Table 5).

Echocardiographic phenomena have been very rarely analyzed in terms of their impact on long-term survival after TLE A couple of papers showed only the role of so-called ghosts, i.e., post-removal, tubular, mobile masses following the lead’s intracardiac route in the right-sided heart chambers [15,20]. The present study set out to investigate the usefulness of preoperative transesophageal echocardiography in the assessment of the long-term survival after TLE. The results are consistent with those observed in earlier studies [5,6,7,8,9,10,11,12,13,14,15,16,17,18,19,20,21,22] which showed that patient-dependent variables (demographic, related to the underlying disease, comorbidities, systemic infection) were the main risk factors for death at long-term follow-up. CIED-related factors, including the number of leads, left ventricular lead and ICD lead play a significant role, but secondary to the underlying condition [5,6,11,12,13,14,15,19,21]. Of the previously identified echocardiographic factors [7,12,16], this study confirms the prognostic value of left ventricular ejection fraction. It should, however be emphasized that the present study was designed to investigate the role of the factors that have not been considered in previous analyses of long-term survival after TLE. Because of the complexity of relationships the preoperative TEE findings and abnormalities were divided into: (1) Non-modifiable factors related to the patient’s general condition; (2) non-modifiable factors related to the underlying disease (indication-dependent); (3) factors that have no effect on the procedure course and chances of long-term survival; (4) factors that may increase the complexity of the extraction procedure and the development of complications but which per se do not decrease chances of long-term survival; and (5) abnormalities that can be corrected during the extraction procedure.

1. Non-modifiable factors related to the patient’s general condition:
  Tricuspid valve regurgitation (TVR), excluding LDTD, was associated with worse long-term survival in multivariate analysis, but after adjustment for common risk factors for poor prognosis this variable lost its prognostic value. In patients referred for lead extraction TVR is a non-modifiable factor because of right ventricular status.
2. Non-modifiable factors related to the underlying disease (indication-dependent):
  Vegetations and LRIE have always been (in all previous analyses, including ours) one of the most potent factors decreasing chances of long-term survival [5,6,7,8,9,10,11,12,13,14,15,16,17,18,19,20,21,22]. Unfortunately, despite the improving standards [2,3,4] long-term mortality among patients after TLE performed due to LRIE does not improve as desired.
3. Factors that have no effect on the procedure course and chances of long-term survival: AMEL (clots, vegetation-like masses) had no influence on chances of long-term survival in our analysis.
4. Factors that may increase the complexity of the extraction procedure and the development of complications but which, per se, do not decrease chances of long-term survival: fibrous tissue binding the lead to the heart structures, lead-to-lead adhesion. The degree of connective tissue overgrowth in response of the endothelium to long-term irritation by the lead depends on implant duration, stiffness and the number of leads, but first of all on patient age (inverse relationship). This phenomenon has been better documented in papers describing lead removal in children and young patients and in adults with leads implanted in childhood [17]. Surprisingly, the current study found that various forms of connective tissue overgrowth (fibrous tissue binding the lead to the heart structures, lead-to-lead adhesion, lead thickening, scar tissue surrounding the lead) were associated with better long-term survival, although based on previous observations [31,32], connective tissue overgrowth was a predictor of TLE technical difficulty and major complications. This proves that the course of the procedure does not affect prognosis after TLE.
5. Abnormalities that can be corrected during the extraction procedure:
  - Lead-dependent tricuspid dysfunction was not significantly associated with the length of survival. This can be attributed to the fact that most patients with LDTD were referred for the intervention because of the lead propping one of the leaflets open, which was corrected to varying extent during TLE.
  - Excessive lead loops (loop in the right atrium, loop crossing the TV, loop in the right ventricle or pulmonary artery) in univariate and multivariate Cox analysis were significantly associated with better survival odds. The reason was that this abnormality was the indication (main or accompanying) for lead replacement and no patient left our facility with abandoned leads.
  - Perforation, penetration—as was the case of lead loops, perforation/penetration was the main or accompanying indication for lead extraction (most of them were “dry” and caused lead dysfunction or, less frequently, it was an incidental finding or the cause of fluid accumulation in the epicardial space). All perforating/penetrating leads were replaced, thus eliminating their influence on survival and future quality of life. Similar to lead loops, perforations in univariate and multivariate Cox analysis of Model 1 were significantly related to better survival odds.

## 6. Limitations

The current study is a single center observational prospective study. The lead extraction procedure was performed using mechanical tools and not laser sheaths.

## 7. Conclusions

The main factors predicting shorter survival among patients undergoing TLE were those related to the patient (patient age during TLE, male gender, higher NYHA class, low LVEF, atrial fibrillation, or chronic renal failure), and those related to the underlying disease, comorbidities and systemic infection.

Non-modifiable factors (patient-dependent and indication/infection-dependent) may have a negative influence on the postoperative course and long-term survival.

The exacerbation of the foreign body reaction resulting in fibrous tissue binding the lead to the vena cava superior or heart structures (especially right atrium wall) seemingly improves chances of longer survival.

## Figures and Tables

**Table 1:** Preliminary analysis of all parameters as potential risk factors for early death after TLE.

	All Group *n* = 936	Alive *n* = 824	Death *n* = 112	“U” Mann–Whitney/ Χ^2^ Test
Follow-up (days); mean ± SD; (min.-max.); median, [Q_1_; Q_3_]	566.2 ± 224.5 (2–730) 730.0 [397.0; 730.0]	604.4 ± 196.0 (64–730) 730.0 [505.0; 730.0]	285.3 ± 221.6 (2–725) 242.0 [75.0; 450.5]	*p* < 0.001
Demographic and clinical data				
Patient age during TLE (years); mean ± SD; median, [Q_1_; Q_3_]	67.08 ± 14.50 69.20 [61.10; 77.80]	66.07 ± 4.79 69.40 [60.60; 77.40]	73.29 ± 10.54 73.30 [66.70–82.20]	*p* < 0.001
Patient age at first CIED implantation (years); mean ± SD; median, [Q_1_; Q_3_]	57.28 ± 16.09 60.40 [50.20; 68.10]	56.32 ± 16.45 60.80 [49.90; 68.20]	64.38 ± 10.85 65.30 [59.10; 72.80]	*p* < 0.001
Sex (female); *n* (%)	355 (37.927)	327 (39.684)	28 (25.000)	*p* = 0.004
NYHA class; (mean ± SD)	2.029 ± 0.574	1.985 ± 0.560	2.348 ± 0.581	*p* < 0.001
LVEF (%); (mean ± SD); median, [Q_1_; Q_3_]	47.89 ± 15.56 53.00 [34.50; 60.20]	48.97 ± 15.05 55,40 [35.00; 60.10]	39.94 ± 16.96 37.00 [25.70; 55.30]	*p* < 0.001
Atrial Fibrillation; *n* (%)	219 (23.397)	177 (21.279)	42 (37.500)	*p* < 0.001
Chronic kidney disease (creatinine concentration >1.3 mg/dL); *n* (%)	230 (24.892)	173 (21.297)	57 (51.351)	*p* < 0.001
Diabetes (any); *n* (%)	198 (21.154)	167 (20.267)	31 (27.679)	*p* = 0.093
CABG history; *n* (%)	74 (7.906)	65 (7.888)	9 (8.036)	*p* = 0.895
Previous sternotomy; *n* (%)	132 (14.103)	116 (14.078)	16 14.286)	*p* = 0.932
Arterial hypertension; n (%)	482 (51.496)	433 (52.549)	49 (43.750)	*p* = 0.100
CHA2DS2-VAsc; mean ± SD	3.005 ± 1.487	2.914 ± 1.763	3.682 ± 1.502	*p* < 0.001
Charlson comorbidity index; mean ± SD	4.886 ± 3.764	4.674 ± 3.751	6.555 ± 3.557	*p* < 0.001
Need for long-term anticoagulation; *n* (%)	389 (41.560)	323 (39.199)	66 (58.929)	*p* < 0.001
Need for long-term antiplatelet therapy; *n* (%)	424 (45.299)	365 (44.296)	59 (52.679)	*p* = 0.116
CIED-related data				
Number of leads in the system before TLE; mean ± SD	1.834 ± 0.639	1.817 ± 0.611	1.946 ± 0.745	*p* = 0.122
Presence of abandoned lead before TLE; *n* (%)	86 (9.188)	74 (8.981)	11 (9.821)	*p* = 0.908
Presence of HV therapy (ICD) lead; *n* (%)	296 (31.624)	249 (30.218)	47 (41.964)	*p* = 0.016
Presence of CS (LV pacing) lead; *n* (%)	153 (16.346)	120 (14.563)	33 (29.464)	*p* < 0.001
Number of leads in the patient before TLE; mean ± SD	1.954 ± 0.729	1.934 ± 0.718	2.098 ± 0.718	*p* = 0.040
Dwell time of the oldest lead in the patient (months); mean ± SD; median, [Q_1_; Q_3_]	115.80 ± 77.6 99.00 [62.00; 156.00]	117.01 ± 77.75 99.00 [64.00; 156.00]	107.24 ± 76.77 84.00 [49.00; 152.00]	*p* = 0.066
Number of procedures before lead extraction; mean ± SD	1.837 ± 0.990	1.840 ± 0.997	1.795 ± 0.922	*p* = 0.766
LRIE certain or probable with or without pocket infection; *n* (%)	151 (16.132)	108 (13.107)	43 (38.393)	*p* < 0.001
Local (pocket) infection (only); *n* (%)	58 (6.196)	52 (6.311)	6 (5.357)	*p* = 0.854
TLE efficacy and complications				
Major complications (any); *n* (%)	18 (1.923)	17 (2.063)	1 (0.893)	*p* = 0.632
Hemopericardium; *n* (%)	12 (1.282)	11 (1.353)	1 (0.893)	*p* = 0.954
Tricuspid severe valve damage during TLE; *n* (%)	6 (0.641)	5 (0.607)	1 (0.893)	*p* = 0.783
Rescue cardiac surgery; *n* (%)	11 (1.175)	10 (1.214)	1 (0.893)	*p* = 0.864
Lack of radiological success; *n* (%)	6 (0.641)	5 (0.607)	1 (0.893	*p* = 0.783
Complete clinical success; (%)	916 (97.863)	805 (97.694)	111 (99.107)	*p* = 0.534
Complete procedural success; *n* (%)	917 (97.761)	808 (98.058)	109 (97.321)	*p* = 0.872

Abbreviations: Q_1_—first quartile, Q_3_—third quartile, CABG—coronary artery bypass grafting, CHA2DS2-VAsc—Score for Atrial Fibrillation Stroke Risk, CIED—cardiac implantable electronic device, CS—coronary sinus, HV—high voltage, LRIE—lead-related infective endocarditis, LV—left ventricle, TLE—transvenous lead extraction.

**Table 2:** Preoperative TEE findings and preliminary evaluation of their potential influence on long-term survival.

Echocardiographic Findings before Transvenous Lead Extraction		All Group	Alive	Death	“U” Mann–Whitney/Χ^2^ Test
		936	824	112	
Tricuspid valve dysfunction (degree of regurgitation)—excluding patients with lead-dependent TV dysfunction	Average tricuspid valve regurgitation (0–4 degree) mean ± SD	1.454 ± 0.956	1.413 ± 0.910	1.759 ± 1.210	*p* < 0.001
	Patients with severe tricuspid regurgitation (3–4) *n* (%)	162 (18.493)	127 (15.423)	35 (31.250)	*p* < 0.001
Lead-dependent tricuspid dysfunction (LDTD)	Average LDTD (0–4) mean ± SD	3.541 ± 0.594	3.500 ± 0.580	3.727 ± 0.647	*p* = 0.212
	Patients with LDTD (any) *n* (%)	60 (6.410)	49 (5.947)	11 (9.821)	*p* = 0.172
	Patients with severe LDTD (3–4) *n* (%)	58 (96.667)	48 (5.825)	10 (8.929)	*p* = 0.285
Any shadows on leads	Patients with any shadows on leads before TLE *n* (%)	607 (64.850)	528 (64.078)	79 (70.536)	*p* = 0.216
Patients with fibrous tissue binding the lead to the SVC and heart structures *n* (%)		236 (25.214)	218 (26.456)	18 (16.071)	*p* = 0.024
Fibrous tissue binding the lead to the superior vena cava and heart structures	Fibrous tissue binding the lead to the heart structures (all) *n* (%)	317 (33.868)	292 (35.436)	25 (22.231)	*p* = 0.008
	Fibrous tissue binding the lead to the SVC *n* (%)	56 (5.983)	53 (6.432)	3 (2.679)	*p* = 0.174
	Fibrous tissue binding the lead to the RA wall *n* (%)	65 (6.944)	65 (7.888)	0 (0.000)	*p* < 0.001
	Fibrous tissue binding the lead to the tricuspid apparatus *n* (%)	90 (9.615)	80 (9.709)	10 (8.929)	*p* = 0.927
	Fibrous tissue binding the lead to the RV wall *n* (%)	106 (11.325)	94 (11.408)	12 (10.714)	*p* = 0.953
Lead-to-lead adhesion *n* (%)		172 (18.377)	162 (19.660)	10 (8.929)	*p* = 0.009
Patients with asymptomatic masses on endocardial leads (AMEL) (patient analysis) *n* (%)		437 (46.688)	391 (47.451)	46 (41.071)	*p* = 0.243
Asymptomatic masses on endocardial leads (AMEL) (analysis)	Lead mass (AMEL) (all) *n* (%)	549 (58.654)	495 (60.007)	54 (48.214)	*p* = 0.022
	Fibrous tissue encasing the lead *n* (% of all AMEL/% of all pts)	160 (29.144/17.094)	150 (30.303/18.204)	10 (18.519/8.929)	*p* = 0.021
	Lead thickening *n* (% of all AMEL/% of all pts)	277 (50.455/29.594)	247 (49.899/29.976)	30 (55.556/26.786)	*p* = 0.560
	Clot on the lead *n* (% of all AMEL/% of all pts)	75 (13.661/8.013)	67 (13.535/8.131)	8 (14.815/7.143)	*p* = 0.860
	Vegetation-like masses *n* (% of all AMEL/% of all pts)	37 (6.740/3.953)	31 (6.263/3.762)	6 (11.111/5.357)	*p* = 0.579
Presence of vegetations (TTE or and TEE)	Patients with vegetations *n* (%)	119 (12.727)	86 (10.450)	33 (29.464)	*p* < 0.001
Excessive lead loops	Patients with lead loops in the heart (any) *n* (%)	181 (19.338)	169 (20.510)	12 (1.7147)	*p* = 0.037
	Lead loops in the RA *n* (%)	138 (14.744)	128 (25.859)	10 (8.929)	*p* = 0.088
	Lead loops in the TV *n* (%)	35 (3.793)	34 (4.126)	1 (0.893)	*p* = 0.154
	Lead loops in the RV or PA *n* (%)	28 (2.991)	27 (3.277)	1 (0.893)	*p* = 0.274
Perforation or penetration of the lead through the cardiac wall up to the epicardium *n* (%)		151 (16.132)	141 (17.112)	10 (8.929)	*p* = 0.038

Abbreviations: AMEL—asymptomatic masses on endocardial leads, LDTD—lead-dependent tricuspid dysfunction, PA—pulmonary artery, RA—right atrial, RV—right ventricular, SVC—superior vena cava, TEE—transesophageal echocardiography, TTE—transthoracic echocardiography.

**Table 3:** Prognostic value of preoperative TEE findings after a follow-up of two years in TLE patients, results of univariate and multivariate model-1 Cox regression analysis.

	Univariate Cox Regression			Multivariate Cox Regression		
	HR	95% CI	*p*	HR	95% CI	*p*
Lead-dependent TV dysfunction (LDTD) (yes/no)	1.630	0.875–3.037	0.124			
TV dysfunction unrelated to lead presence (all) (by one degree)	1.528	1.296–1.801	<0.001	1.481	1.261–1.740	<0.001
Asymptomatic masses on endocardial leads (AMEL) (yes/no)	0.821	0.563–1.196	0.304			
Fibrous tissue encasing the lead (yes/no)	0.442	0.231–0.847	0.014	0.587	0.304–1.132	0.112
Lead thickening (yes/no)	0.911	0.600–1.385	0.664			
Clot on the lead (yes/no)	1.017	0.496–2.089	0.962			
Vegetation-like masses (yes/no)	1.495	0.657–3.404	0.338			
Strong connective tissue scar binding the lead to heart structures (any) (yes/no)	0.624	0.381–1.022	0.061			
Fibrous tissue binding the lead to the SVC (yes/no)	0.414	0.131–1.303	0.132			
Fibrous tissue binding the lead to the SVC or/and RA wall (yes/no)	0.208	0.066–0.655	0.007	0.285	0.088–0.919	0.036
Fibrous tissue binding the lead to the tricuspid apparatus (yes/no)	0.944	0.493–1.807	0.861			
Fibrous tissue binding the lead to the RV wall (yes/no)	0.952	0.523–1.733	0.872			
Fibrous tissue binding the lead to the tricuspid apparatus or/and RV wall (yes/no)	0.886	0.529–1.485	0.646			
Lead-to-lead adhesion (yes/no)	0.484	0.260–0.901	0.022	0.653	0.345–1.235	0.190
Perforation or penetration of the lead through the cardiac wall up to the epicardium (yes/no)	0.474	0.247–0.907	0.024	0.496	0.259–0.953	0.035
Excessive lead loops in the heart (yes/no)	0.543	0.310–0.956	0.033	0.528	0.301–0.928	0.026
Presence of vegetations (yes/no)	3.078	2.042–4.639	<0.001	2.631	1.738–3.983	<0.001

Abbreviations: LDTD—Lead dependent tricuspid valve dysfunction, RV—right ventricle, SVC—superior vena cava, TV—tricuspid valve.

**Table 4:** Prognostic value of TEE findings after a follow-up of two years in TLE patients after adjustment of the Cox regression model for common risk factors for poor prognosis, results of univariate and multivariate model-2 Cox regression analysis.

	Univariable Cox Regression			Multivariable Cox Regression		
	HR	95% CI	*p*	HR	95% CI	*p*
Lead-dependent TV dysfunction (LDTD) (yes/no)	1.630	0.875–3.037	0.124			
TV dysfunction unrelated to lead presence (all) (by one degree)	1.528	1.296–1.801	<0.001	1.128	0.937–1.357	0.202
Asymptomatic masses on endocardial leads AMEL (yes/no)	0.821	0.563–1.196	0.304			
Fibrous tissue encasing the lead (yes/no)	0.442	0.231–0.847	0.014	0.629	0.323–1.226	0.174
Lead thickening (yes/no)	0.911	0.600–1.385	0.664			
Clot on the lead (yes/no)	1.017	0.496–2.089	0.962			
Vegetation-like masses (yes/no)	1.495	0.657–3.404	0.338			
Strong connective tissue scar binding the lead to heart structures (any) (yes/no)	0.624	0.381–1.022	0.061	1.531	0.841–2.787	0.164
Fibrous tissue binding the lead to the SVC (yes/no)	0.414	0.131–1.303	0.132			
Fibrous tissue binding the lead to the SVC or/and RA wall (yes/no)	0.208	0.066–0.655	0.007	0.270	0.077–0.944	0.040
Fibrous tissue binding the lead to the tricuspid apparatus (yes/no)	0.944	0.493–1.807	0.861			
Fibrous tissue binding the lead to the RV wall (yes/no)	0.952	0.523–1.733	0.872			
Fibrous tissue binding the lead to the tricuspid apparatus or/and RV wall (yes/no)	0.886	0.529–1.485	0.646			
Lead-to-lead adhesion (yes/no)	0.484	0.260–0.901	0.022	0.607	0.313–1.175	0.138
Perforation or penetration of the lead through the cardiac wall up to the epicardium (yes/no)	0.474	0.247–0.907	0.024	0.562	0.289–1.093	0.090
Excessive lead loops in the heart (yes/no)	0.543	0.310–0.956	0.033	0.632	0.350–1.139	0.127
Presence of vegetations (LRIE with vegetations) (yes/no)	3.078	2.042–4.639	<0.001	2.613	1.635–4.176	<0.001
Female gender (yes/no)	0.542	0.355–0.826	0.004	0.812	0.511–1.289	0.376
Patient age at first CIED implantation (↑ by 1 year)	1.040	1.023–1.056	0.000	0.997	0.964–1.031	0.871
Patient age during TLE (↑ by 1 year)	1.044	1.026–1.062	0.000	1.037	0.999–1.076	0.057
Need for long-term anticoagulation (yes/no)	2.172	1.490–3.165	0.000	1.472	0.979–2.214	0.063
LVEF (↓by 10%p)	1.420	1.261–1.597	0.000	1.168	0.999–1.360	0.051
NYHA class (↑by one class)	2.852	2.120–3.838	0.000	1.340	0.909–1.975	0.139
Chronic renal failure (yes/no)	3.528	2.435–5.111	0.000	1.811	1.213–2.704	0.004
Diabetes t. 2 (yes/no)	1.522	1.006–2.303	0.047	1.209	0.780–1.874	0.397
Presence of CS lead before TLE (yes/no)	2.342	1.560–3.516	0.000	1.610	1.045–2.482	0.031
Presence of ICD lead before TLE (yes/no)	1.649	1.133–2.401	0.009	0.930	0.573–1.509	0.768
Arterial hypertension (yes/no)	0.727	0.500–1.056	0.094	0.569	0.381–0.849	0.006
Lead-related infective endocarditis (LRIE) without vegetations (yes/no)	2.289	1.196–4.383	0.012	2.371	1.166–4.821	0.017
Isolated local pocket infection without general infection (yes/no)	0.842	0.370–1.915	0.681			

Abbreviations: RV—right ventricle, SVC—superior vena cava, TV—tricuspid valve CIED—cardiac implantable electronic device, CS—coronary sinus, ICD—implantable cardioverter- defibrillator, LVEF—left ventricular ejection fraction, NYHA class—New York Heart Association class, TLE—transvenous lead extraction.

**Table 5:** Potential risk factors for mortality after TLE during long-time follow-up.

Sources	Potential Patient- and Co-Morbidity-Related Risk Factors (Normal Type)					
	*Potential Infection-Related Risk Factors (Italics)*					
	POTENTIAL CIED-, PREVIOUS PROCEDURE- AND TLE-RELATED RISK FACTORS (Capital Letters)					
Author	Year	Patients	Most Important Factor	Important Factor	New Observation	No. of Refer.
Maytin	2012	985	Elderly pts, *Infections*	Diabetes, renal failure	SYSTEM UPGRADE	[5]
Deharo	2012	197 Inf	Age, *Infection, disease-related factors*	Renal failure	*Thrombocytopenia,* CRT	[6]
Habib	2013	415	*Endocarditis*, heart failure	Renal failure	Co-morbidities	[7]
Deckx	2014	176 Inf	*Systemic infection*, female sex	Renal failure	*Low hemoglobin*	[8]
Kim	2014	80 IE	*Valvular endocarditis & MRSA infection*		*MRSA infection*	[9]
Tarakji	2014	502	*Systemic infection, concurrent infection*	Renal *failure*	NYHA III/IV	[10]
Fu	2015	652	*Endocarditis*	“will be reported”	ABANDONED LEAD(?)	[11]
Merchant	2015	508	LVEF, LEAD NUMBER	Renal failure	PROCEDURAL FAILURE	[12]
Gomes	2016	510	*Systemic infection,* advanced age	Renal failure	RETAINED LEAD FRAGMENT, MC	[13]
Gomes	2016	348	*Endocarditis*		RETAINED LEAD FRAGMENT	[14]
Narducci	2016	217	*Endocarditis, systemic infection*		*“Ghost” presence*	[15]
Kutarski	2016	2049	EF, NYHA class, AF	Renal failure, diabetes	LACK OF CLINICAL SUCCESS, CRT-P	[16]
Diemberger	2018	169	*Infection*	*Presence of vegetations (TEE!!!)*	Risk factors for development of CIEDI (Shariff score ≥3)	[17]
Diemberger	2019	105 CIEDI	*Endocarditis*	*18F-FDG PET/CT imaging*	*Endocarditis without pocket infection*	[18]
Polewczyk	2016	500 IE	*Vegetation size*, ICD LEAD	Renal failure, AF	*Vegetation remnant, hemoglobin*	[19]
Diemberger	2018	121		Renal failure	*“Ghost” presence & closed pocket & modified Duke criteria fulfilled*	[20]
Jacheć	2017	1884	*CIEDI & pocket infection*	Renal failure, age, diabetes, infection (any)	ICD LEAD	[21]
Seifert	2018	537	*Staph aureus*	Renal failure, age,	N-terminal pro B-type natriuretic peptide level ≥3000 pg/mL, AF	[22]
Zucchelli	2019	3555	RIATA LEAD OCCLUSION OF SUPERIOR VENOUS ACCESS UTILITY OF POWERED ONES	Notice: only for acute outcome and intrahospital mortality		[34]
Segreti	2019	3555	ABANDONED LEADS	Notice: only for acute outcome		[35]

## Data Availability

Readers can access the data supporting the conclusions of the study at www.usuwanieelektrod.pl.

## Supplementary Materials

The following are available online at https://www.mdpi.com/1660-4601/18/4/1862/s1, Figure S1: Prognostic value of TEE findings in follow-up of 2 years in TLE patients after adjustment of the Cox regression model for common risk factors for poor prognosis, results of multivariable Cox regression analysis.
