# Supplementary material for: Prognostic Value of Preoperative Echocardiographic Findings in Patients Undergoing Transvenous Lead Extraction
Source: Int J Environ Res Public Health. 2021 Feb 14;18(4):1862. doi: 10.3390/ijerph18041862 (PMC7918219; doi:10.3390/ijerph18041862)
Supplement: Supplementary file 1 [file ijerph-18-01862-s001.pdf]

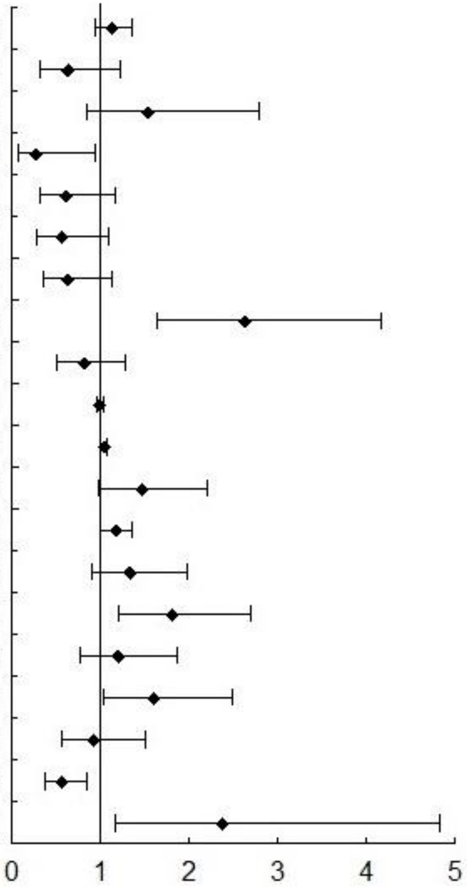

	HR	95%CI	P
TV dysfunction unrelated to lead presence (all) [by one degree]	1,128	0,937-1,357	0,202
Fibrous tissue encasing the lead [yes / no]	0,629	0,323-1,226	0,174
Connective tissue scar binding the lead to heart structures (any) [yes / no]	1,531	0,841-2,787	0,164
Fibrous tissue binding the lead to the SVC or / and RA wall [yes / no]	0,270	0,077-0,944	0,04
Lead-to-lead adhesion [yes / no]	0,607	0,313-1,175	0,138
Perforation or penetration through the cardiac wall up to the epicardium [yes / no]	0,562	0,289-1,093	0,090
Excessive lead loops in the heart [yes / no]	0,632	0,350-1,139	0,127
Presence of vegetations (LRIE with vegetations) [yes / no]	2,613	1,635-4,176	<0,001
Female gender [yes / no]	0,812	0,511-1,289	0,376
Patient age at first CIED implantation [by 1 year]	0,997	0,964-1,031	0,871
Patient age during TLE [by 1 year]	1,037	0,999-1,076	0,057
Need for long-term anticoagulation [yes / no]	1,472	0,979-2,214	0,063
LVEF [decrease by 10%p]	1,168	0,999-1,360	0,051
NYHA class [by one class]	1,34	0,909-1,975	0,139
Chronic renal failure [yes / no]	1,811	1,213-2,704	0,004
Diabetes t. 2 [yes / no]	1,209	0,780-1,874	0,397
Presence of CS lead before TLE [yes / no]	1,610	1,045-2,482	0,031
Presence of ICD lead before TLE [yes / no]	0,930	0,573-1,509	0,768
Arterial hypertension [yes / no]	0,569	0,381-0,849	0,006
Lead-related infective endocarditis (LRIE) without vegetations [yes / no]	2,371	1,166-4,821	0,017

Figure S1. Prognostic value of TEE findings in follow-up of 2 years in TLE patients after adjustment of the Cox regression model for common risk factors for poor prognosis, results of multivariable Cox regression analysis.
